# Toxicological Evaluation of Ionic Liquids: QSAR Approach for Acetylcholinesterase Enzyme Inhibition

**DOI:** 10.1021/acs.chemrestox.5c00475

**Published:** 2026-02-17

**Authors:** Ali Ebrahimpoor Gorji, Petri Uusi-Kyyny, Ville Alopaeus

**Affiliations:** School of Chemical Technology, Department of Chemical and Metallurgical Engineering, Research Group of Chemical Engineering, Aalto University, P.O. Box 16100, FI-00076 Aalto, Finland

## Abstract

A “quantitative structure–activity relationship” (QSAR) model is developed to predict the toxicity of ionic liquids (ILs) based on the effect on the acetylcholinesterase (AChE) enzyme. A data set of 243 ILs was compiled and randomly divided into training (183 ILs) and test (60 ILs) sets to enable both internal and external validations. To optimize the model performance, a breaking point analysis was performed to identify the most relevant molecular descriptors. The analysis revealed that a set of 11 COSMO-RS quantum chemical descriptors provided near-optimal predictive power, with additional descriptors offering minimal improvement. A multiple linear regression (MLR) model was developed by using these descriptors, incorporating both cationic and anionic molecular features. Internal validation using Leave-One-Out and Leave-Many-Out cross-validation (*Q* ^2^_LOO_ = 0.79, *Q* ^2^_LMO_ = 0.78) as well as Y-scrambling confirmed the robustness of the model. External validation on the test set yielded acceptable *R* ^2^ = 0.75 and low RMSE = 0.35 values, indicating strong predictive performance. The developed model outperformed previous models, particularly by accounting for the influence of anion structures, which have been largely neglected in earlier works. The final MLR-QSAR model not only demonstrated statistical reliability but also provided mechanistic insights into the structural contributions of both ionic components to IL’s toxicity. Predicted toxicity values (Log 1/EC50) for novel ILs are also presented, expanding our understanding of IL safety profiles.

## Graphical abstract

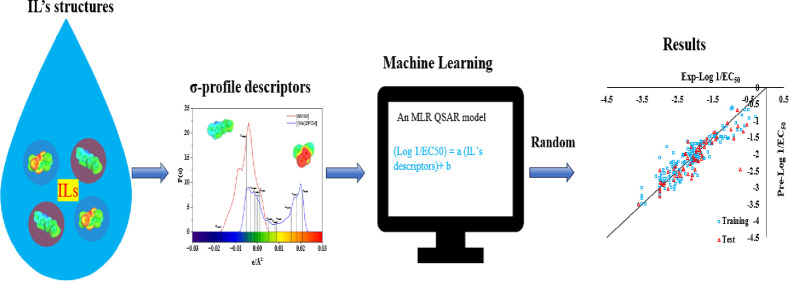

## 1 Introduction

Ionic liquids (ILs) have emerged as promising alternatives to conventional solvents due to their exceptional physicochemical characteristics, including negligible vapor pressure, thermal stability, structural tunability, and low flammability.^1^ Composed of organic cations paired with either organic or inorganic anions, ILs offer a vast range of possible combinations, enabling customization for specific industrial and research applications such as green chemistry and energy systems.^2^ Low vapor pressure of ILs is often assumed to lead automatically to greener chemicals than other industrially used solvents, as ILs are essentially nonvolatile. The mere number of possible anion–cation combinations in IL synthesis is considered proof that their potential as solvents is limitless, as there must be a combination that is optimal for each application. Neither of these claims is completely true: the structural flexibility presents huge challenge as experimental screening of all potential IL candidates is simply impossible. Low vapor pressure is also only one aspect among many when assessing how green a solvent is. Other very important green solvent properties, such as their environmental safety and biological impact, are often overlooked. As the number of newly synthesized ILs continues to grow, careful assessment of their toxicity has become increasingly important.

In one of the most comprehensive experimental studies to date by Ranke et al.,^3^ toxicity experimental data for a limited number of ILs were reported, including effective concentration 50% (EC50) values for 253 compounds against the rat leukemia cell line IPC-81 (Institute of Public Health Cytotoxicity-81) and acetylcholinesterase (AChE, acetylcholinesterase enzyme) inhibition for 292 compounds. Despite the value of this data set, it represents only a fraction of the vast chemical space of ILs. Given the almost limitless possibilities for generating new ILs through permutations of different cationic and anionic species, the number of experimentally evaluated compounds remains insufficient. Many potentially useful ILs have no available toxicity data, underscoring the urgent need for predictive toxicology approaches to bridge this knowledge gap and guide the rational design of a safer IL. It is important to note that a lower EC_50_ indicates a higher toxicity of the IL.

In recent years, different machine-learning (ML) algorithms such as multiple linear regression (MLR), neural networks (NN), random forest (RF), cascade correlation network (CCN), extreme learning machine (ELM), supporting vector machine (SVM), and extreme boosting (X-Boosting) have been employed to predict IPC-81 and AChE.^4–16^ A comprehensive review of the literature was conducted, focusing on these modeling approaches across various classes of ILs and related compounds, with key findings summarized in Table 1.

**Table 1:** Number of Data Points, Type of Descriptors Used and Different Used ML Algorithms for the Prediction of Toxicity of ILs against IPC-81 or AChE and Reported Values of Statistical Parameters

research group (year)	number of data points	type and number of used descriptors (inputs)	applied ML algorithms	studied targets	best reported values of statistical parameters		ref
					***R*** ^***2***^	**RMSE**	
Sadaghiyanfam et al. (2025)	355	More than 10 selected descriptors from ChemBERTa embeddings, Fingerprint, molecular descriptors	NN, X-boosting, SVM	IPC-81	0.86	0.3899	^4^
Wu et al. (2024)	160	14 ***cationic*** descriptors (without ***anionic*** descriptors) were selected from 244 topological, constitutional, and hybrid descriptors	RF and X-boosting	**AChE**	0.85	0.1500	^5^
Wang et al. (2021)	355	42 group contribution-descriptors from RDKit cheminformatics tool	NN and SVM	IPC-81	0.92	0.2875	^6^
Yan et al. (2021)	153	MOE and Dragon descriptors	RF, Nearest Neighbor, X-boosting, NN	**AChE**	0.88	0.1700	^7^
Zhu et al. (2019)	160	11 ***cationic*** descriptors (without any ***anionic*** descriptors), electrostatic potential surface area and charge density distribution area descriptors	MLR and Extreme Learning Machine (ELM)	**AChE**	0.91	0.1450	^8^
Cao et al. (2018)	119	8 electrostatic potential surface area and charge distribution area	MLR, SVM, and ELM	IPC-81	0.97	0.1570	^9^
Cho and Yun (2016)	251	6 descriptors from a pool including 8 linear free-energy relationship (LFER) descriptors	MLR	**AChE**	0.74		^10^
Basant et al. (2015)	232	4 selected descriptors from a pool including 211 Moses-descriptors (physicochemical, constitutional, geometrical, topological, and spatial)	CCN and SVM	**AChE**	0.97	0.1000	^11^
Peric et al. (2015)	55	10 group contribution descriptors	MLR	IPC-81 and **AChE**	0.91		^12^
Das and Roy (2013)	232	11 ***cationic*** descriptors (without any ***anionic*** descriptors), none-ETA and atomic fingerprint descriptors	MLR (Partial Least Squares (PLS))	**AChE**	0.83	0.2430	^13^
Zhao et al. (2014)	100	CODESSA descriptors	MLR and SVM	IPC-81	0.95	0.2340	^14^
Yan et al. (2012)	221	17 descriptors including topological index and cation atom number	MLR	**AChE**	0.87		^15^
Torrecilla et al. (2009)	153	12 descriptors from a pool including 46 constitutional descriptors	MLR, Multilayer perceptron (MLP), and radial-basis function (RB)	IPC-81 and **AChE**	0.93		^16^

As can be seen in Table 1, in previous studies assessing the toxicity of ILs, two primary targets have been commonly used: IPC-81 and AChE, with this work focusing primarily on AChE inhibition. A critical review of the literature and data summarized in Table 1 reveals several methodological issues that remain to be addressed, particularly in the design of predictive models for IL’s toxicity. One major concern is that several studies have built predictive models using only cationic descriptors despite substantial evidence that anions also contribute to the toxicity of ILs. Some studies have constructed linear models incorporating both cationic and anionic descriptors, highlighting the importance of considering contributions from both ions.^10,15^ Although the effect of the anion has been discussed less extensively than that of the cation, available evidence indicates that ionic liquid toxicity can change significantly upon variation of the anion while the cation is kept constant. For example, there is a clear difference between the measured toxicities for some ILs, such as ([1-butyl-3-methylimidazolium] [trifluoridotris­(pentafluoroethyl)­phosphate] (IL-83) and [1-butyl-3-methylimidazolium] [bis­(trifluoromethyl)­amide] (IL-198). However, in many linear^8,13^ and even nonlinear modeling studies, such as those employing RF,^5^ X-boosting,^5^ and ELM,^8^ only cationic descriptors were used for modeling the toxicity. These models face a significant limitation as they are unable to distinguish the impact of different anion structures on toxicity. As a result, they predict the same toxicity for all ILs that share the same cations but have different anions. These models demonstrated good fitting performance, possibly due to the limited structural diversity in their data set. Specifically, in cases where the cationic moiety remained constant and only the anions varied, the experimental AChE toxicity values were quite similar, which might have enabled the models to fit well even without the inclusion of anionic descriptors. Nonetheless, this raises concerns. It is known from the literature that quantitative AChE toxicity data are currently available for around 251 ILs, but these studies only used 160 ILs, which calls into question the representativeness and generalizability of their models. Additionally, a study[Bibr ref13] (2013) used a linear model with 11 cationic descriptors only to predict the toxicity of 232 ILs, but the model’s performance was notably lower compared to the aforementioned nonlinear models.^5,8^ This result is not surprising, as it combined a larger and possibly more diverse data set with a relatively simple modeling technique. Notably, that study also showed that for ILs with identical cations but different anions, the model predicted the same toxicity values despite significant differences in their experimental AChE inhibition data. This further supports the hypothesis that excluding anionic descriptors from the modeling process can reduce the predictive accuracy. In summary, to capture the observed structure–toxicity relationships of ILs, especially for AChE inhibition, it is essential that both cationic and anionic descriptors be included in the final predictive models.

Another critical issue observed in previous studies concerns the type and number of molecular descriptors, both cationic and anionic, used for developing predictive models, particularly in relation to the number of ILs included in the data set (i.e., number of data points). It is evident that both the diversity and quality of descriptors, as well as their balance with the sample size, can significantly influence the model performance and generalizability. For instance, in one study utilizing a small descriptor pool including 8 linear free-energy relationship (LFER) descriptors,^10^ a subset of 6 cationic and anionic descriptors was selected to model a data set of 251 ILs for which experimental AChE toxicity values were available. Despite this relatively large and informative data set, the resulting linear model did not achieve satisfactory accuracy. This limitation highlights the importance of exploring more advanced or higher-dimensional descriptors, such as quantum chemical descriptors, which can provide a richer and more comprehensive feature space. These descriptors offer the potential to improve model performance, especially when paired with nonlinear modeling techniques. Quantum descriptors have been employed in some nonlinear modeling efforts, such as in the work by Zhu et al.,^8^ though applied to a smaller data set of 160 ILs. This suggests the potential of these descriptors but also points to the need for larger and more diverse data sets to fully leverage their benefits. Moreover, some studies have raised concerns about the inadequate ratio of descriptors to data points, which can lead to overfitting or unreliable generalization. For example, Torrecilla et al. (2009)^16^ used 12 constitutional descriptors to model the toxicity of just 153 ILs, while Perić et al. (2015)^12^ employed 10 group contribution-based descriptors for a data set of only 55 ILs. In both cases, the descriptor-to-sample ratio was arguably too high, potentially compromising the robustness of the models despite the seemingly high reported statistical metrics. It is therefore crucial to maintain an appropriate descriptor-to-sample ratio and to carefully select descriptors based not only on theoretical relevance but also on their ability to represent structural diversity without overfitting. Future research should focus on constructing larger and more balanced data sets and employing descriptor sets that capture both electronic and structural properties, especially when using advanced ML techniques.

The use of Quantitative Structure–Activity Relationship (QSAR) modeling has emerged as a robust strategy for uncovering the intricate relationship between molecular structures and activity,^17,18^ such as IL’s toxicity. Unlike some studies summarized in Table 1, the QSAR framework provides an extended capability to analyze a broader array of molecular features by computing and evaluating a comprehensive set of structural descriptors. One critical, yet often overlooked, step in this process is the rational selection of descriptors from a high-dimensional descriptor space (or descriptor pool), a factor that significantly impacts the interpretability and predictive strength of the final model. This approach not only improves the transparency of the developed models but also contributes to a deeper understanding of molecular influences on IL’s toxicity. To ensure methodological rigor, the QSAR modeling in this study has been performed using QSARINS,^19–21^ a validated platform that incorporates extensive tools for both internal and external model validations, enhancing reliability and predictive confidence.

By developing a QSAR model that integrates both cationic and anionic descriptors, this study provides a powerful predictive tool that not only predicts ionic liquids’ toxicity with high accuracy but also reveals intricate mechanistic insights into their structure–activity relationships. In continuation of efforts to improve the design of ILs, the primary goal of this study is to develop an accurate predictive model for assessing their toxicity, particularly regarding AChE inhibition. Given the high potential of ILs in industrial and environmental applications, yet growing concerns about their biological safety, this work takes advantage of the QSAR approach to identify the molecular features contributing to toxicity. Through this methodology, it becomes possible to rationally design novel IL structures that maintain high performance while exhibiting minimal toxic effects, thus paving the way for the development of much greener and more sustainable solvents. This study, therefore, marks an important step toward the next generation of safer and more environmentally friendly ILs.

## 2 Method

### 2.1 Basic Theory

As a continuation of former studies,^8,10,13,16^ the dependency of IL’s toxicity is related to the IL’s structure using new molecular descriptors in this study. The model of this study is shown as eq 1:

$$\mathrm{Log}\;1/{\mathrm{EC}}_{50}\;\mathrm{in}\;\log\;\mathrm{unit}\;\mathrm{of}\;\mathrm{\mu M}=a_{1}(\mathrm{Cat}\mathrm{Des}{)}_{1}+\cdot \cdot \cdot +a_{n}(\mathrm{Cat}\mathrm{Des}{)}_{n}+b_{1}(\mathrm{Ani}\mathrm{Des}{)}_{1}+b_{m}(\mathrm{Ani}\mathrm{Des}{)}_{m}+c1$$ 1

where *a*, *b*, and *c* are adjustable parameters. *n* and *m* represent the number of used cationic and anionic descriptors in the model, respectively.

### 2.2 Data Set

Drawing from a broad spectrum of previous data sets,^10,11,13,15^ this investigation introduces the data set including 243 ILs (i.e., 243 data points), as summarized in Table 2. The prior most comprehensive data set, utilized for model development and validation,^10^ involved 251 ILs. Details on variations of used cations and anions are available in the Supporting Information (Sheet 1). There are 113 distinct cations and 29 anions. The gathered set of ILs included eight distinct cationic cores: imidazolium (IM), ammonium (N), pyridinium (Py), pyrrolidinium (Pyr), phosphonium (P), piperidinium (Pip), quinolinium (Quin), and morpholinium (Mor). These cation types featured diverse functional moieties and substitutions, along with a range of counter-anions.

**Table 2:** 243-Studied ILs in This Study Alongside Experimental and Predicted Values (Values of the Logarithm of the Inverse Effective Concentration 50% (EC50)) Using eq 13 (This Study) and Former Linear Models in the Literature^a^

no.	ILs	exp log 1/EC_50_	pre-this study	pre by^8^	pre by^10^	pre by^15^
1	1-[(hexyloxy)methyl]-3-hydroxypyridinium 1,1-dioxo-1,2-dihydrobenzo[d]isothiazol-3-onate	–3.60	–3.51		–3.61	
**2**	**1-[(hexyloxy)methyl]-3-hydroxypyridinium acesulfamates**	–3.60	–3.50		–3.24	
3	1-[(heptyloxy)methyl]-3-hydroxypyridinium 1,1-dioxo-1,2-dihydrobenzo[d]isothiazol-3-onate	–3.50	–3.33		–3.58	
4	1-[(heptyloxy)methyl]-3-hydroxypyridinium chloride	–3.50	–3.01		–2.68	
5	1-butoxymethyl-3-hydroxypyridinium acesulfamates	–3.50	–3.24		–3.03	
6	1-butoxymethyl-3-hydroxypyridinium 1,1-dioxo-1,2-dihydrobenzo[d]isothiazol-3-onate	–3.50	–3.25		–3.40	
7	3-hydroxy-1-(propoxymethyl)pyridinium acesulfamates	–3.50	–3.50		–3.06	
8	3-hydroxy-1-(propoxymethyl)pyridinium 1,1-dioxo-1,2-dihydrobenzo[d]isothiazol-3-onate	–3.40	–3.51		–3.18	
9	tetrabutylphosphonium bis[1,2-benzenediolato(2-)-O1,O2]borate	–3.11	–2.67		–2.79	
10	1-butyl-1-methylpyrrolidinium trifluoridotris(pentafluoroethyl)phosphate	–3.00	–2.70		–2.20	
11	1-(3-carboxypropyl)-3-methylimidazolium chloride	–3.00	–3.16		–3.57	
12	(2-hydroxyethyl)dimethylammonium formate	–3.00	–3.21		–3.15	
**13**	**(2-hydroxyethyl)dimethylammonium hydroxyacetate**	–3.00	–3.27		–3.25	
14	(2-hydroxyethyl)trimethylammonium 2-hydroxypropanoate	–3.00	–3.06		–3.13	
**15**	**(2-hydroxyethyl)trimethylammonium hydroxyacetate**	–3.00	–3.10		–3.12	
16	(cyanomethyl)ethyldimethylammonium bis(trifluoromethylsulfonyl)amide	–3.00	–3.02		–3.57	
**17**	**(cyanomethyl)ethyldimethylammonium chloride**	–3.00	–3.03		–3.39	
18	ethyl(3-hydroxypropyl)dimethylammonium bis(trifluoromethylsulfonyl)amide	–3.00	–2.95		–3.27	
**19**	**1-(7-carboxyheptyl)-3-methylimidazolium bromide**	–3.00	–2.48		–3.49	
20	1-methylimidazolium tetrafluoroborate	–3.00	–2.10		–2.28	
21	(2-hydroxyethyl)ammonium formate	–3.00	–3.28		–3.42	
22	1-(3-hydroxypropyl)-3-methylimidazolium chloride	–2.99	–2.81		–2.87	–2.78
**23**	**4-(2-methoxylethyl)-4-methylmorpholinium chloride**	–2.99	–2.84	–2.85	–2.56	–2.97
24	ethyl(3-methoxypropyl)dimethylammonium chloride	–2.97	–2.78	–2.78	–2.70	–2.71
25	4-(ethoxymethyl)-4-methylmorpholinium chloride	–2.96	–2.58	–2.74	–2.52	–2.75
**26**	**4-(2-hydroxyethyl)-4-methylmorpholinium iodide**	–2.96	–2.87	–3.25	–3.35	–2.91
27	1-(2-hydroxyethyl)-3-methylimidazolium iodide	–2.96	–2.67	–2.85	–3.17	–2.78
28	4-(2-hydroxyethyl)-4-methylmorpholinium bis(trifluoromethylsulfonyl)amide	–2.93	–2.87	–3.25	–3.21	–2.91
29	ethyl(3-methoxypropyl)dimethylammonium bis(trifluoromethylsulfonyl)amide	–2.92	–2.78	–2.78	–2.88	–2.71
**30**	**4-(2-methoxyethyl)-4-methylmorpholinium bis(trifluoromethylsulfonyl)amide**	–2.90	–2.92	–2.85	–2.74	–2.97
31	tetraethylammonium Bis[1,2-benzenediolato(2-)-O1,O2]borate	–2.90	–2.48		–3.11	
32	triethylethanaminium bis[1,2-benzenediolato(2-)-O1,O2]borate	–2.90	–2.48		–3.11	
33	1-(cyanomethyl)-3-methylimidazolium chloride	–2.89	–2.89	–2.91	–2.41	–2.80
34	1-(cyanomethyl)-1-methylpyrrolidinium chloride	–2.88	–2.73		–2.41	–2.78
**35**	**1-(cyanomethyl)-3-methylimidazolium bis(trifluoromethylsulfonyl)amide**	–2.88	–2.88	–2.91	–3.06	–2.80
36	4-(ethoxymethyl)-4-methylmorpholinium bis(trifluoromethylsulfonyl)amide	–2.88	–2.57	–2.74	–2.70	–2.76
37	3-(2-hydroxyethyl)-1-methylimidazolium bis(trifluoromethylsulfonyl)imide	–2.88	–2.80	–2.85	–3.02	–2.78
38	1-(3-hydroxypropyl)-1-methylpyrrolidinium chloride	–2.86	–3.03	–2.81	–2.53	–2.77
39	1-(cyanomethyl)-1-methylpyrrolidinium bis(trifluoromethylsulfonyl)amide	–2.83	–2.73		–2.59	–2.78
40	tetraethylammonium chloride	–2.80	–1.77		–2.13	–1.95
41	1-methyl-3-(3-oxobutyl)imidazolium bromide	–2.79	–2.44		–2.30	–3.12
**42**	**4-butyl-4-methylmorpholinium (bis(trifluoromethylsulfonyl)amide)**	–2.78	–2.52	–2.80	–2.14	–2.28
**43**	**1-(3-hydroxypropyl)-1-methylpyrrolidinium bis(trifluoromethylsulfonyl)amide**	–2.77	–3.03	–2.81	–2.71	–2.78
44	1-(3-hydroxypropyl)-3-methylimidazolium bis(trifluoromethylsulfonyl)amide	–2.74	–2.80		–3.05	–2.79
45	1-(3-methoxypropyl)-1-methylpyrrolidinium chloride	–2.74	–2.68	–2.78	–2.21	–2.71
46	4-butyl-4-methylmorpholinium bromide	–2.71	–2.53	–2.80	–2.11	–2.28
47	1-(3-methoxypropyl)-1-methylpyrrolidinium bis(trifluoromethylsulfonyl)amide	–2.71	–2.67	–2.78	–2.39	–2.72
48	1-(4-hydroxybutyl)-3-methylimidazolium chloride	–2.70	–2.93		–2.87	–2.73
49	1-(2-hydroxyethyl)pyridinium iodide	–2.69	–2.26	–2.48	–3.07	–2.62
50	ethyl(2-hydroxyethyl)dimethylammonium iodide	–2.67	–3.00		–3.39	–2.66
51	1-(2-hydroxyethyl)pyridinium bis(trifluoromethylsulfonyl)amide	–2.65	–2.25	–2.48	–2.93	–2.62
52	1-(3-hydroxypropyl)pyridinium chloride	–2.65	–2.38	–2.35	–2.59	–2.53
53	1-(2-hydroxyethyl)-1-methylpyrrolidinium iodide	–2.63	–2.78	–2.60	–2.78	–2.66
**54**	**1-(2-hydroxyethyl)-1-methylpyrrolidinium bis(trifluoromethylsulfonyl)amide**	–2.61	–2.78	–2.60	–2.64	–2.67
55	1-(3-methoxypropyl)-3-methylimidazolium chloride	–2.61	–2.56		–2.42	–2.49
56	tetrabutylphosphonium bromide	–2.61	–1.96		–1.96	–2.61
**57**	**butyltrimethylammonium bis(trifluoromethylsulfonyl)amide**	–2.60	–2.44	–2.40	–2.32	–2.32
58	1-(2-ethoxyethyl)-1-methylpiperidinium bromide	–2.60	–2.30	–2.47	–2.10	–2.22
59	1-hexyl-1-methylpyrrolidinium bis(trifluoromethylsulfonyl)amide	–2.60	–2.22		–2.02	–2.19
60	1-(2-ethoxyethyl)-1-methylpyrrolidinium bromide	–2.60	–2.42	–2.27	–2.17	–2.54
61	Ethyl(2-hydroxyethyl)dimethylammonium bis(trifluoromethylsulfonyl)amide	–2.59	–3.00		–3.24	–2.67
62	4-ethyl-4-methylmorpholinium 4-methylbenzenesulfonate	–2.59	–3.05	–2.41	–2.51	–2.02
**63**	**1-(2-methoxyethyl)-3-methylimidazolium chloride**	–2.58	–2.64	–2.51	–2.33	–2.44
64	1-(3-methoxypropyl)-3-methylimidazolium bis(trifluoromethylsulfonyl)amide	–2.58	–2.56		–2.60	–2.50
65	Ethyl(2-methoxyethyl)dimethylammonium chloride	–2.57	–2.56	–2.28	–2.70	–2.56
66	1-(3-hydroxypropyl)-1-methylpiperidinium bis(trifluoromethylsulfonyl)amide	–2.56	–2.89	–2.66	–2.66	–2.51
67	1-(3-hydroxypropyl)pyridinium bis(trifluoromethylsulfonyl)amide	–2.56	–2.37	–2.35	–2.77	–2.54
**68**	**Ethyl(2-ethoxyethyl)dimethylammonium chloride**	–2.56	–2.36	–2.63	–2.56	–2.57
69	1-(2-Ethoxyethyl)-1-methylpiperidinium bis(trifluoromethylsulfonyl)amide	–2.55	–2.30	–2.47	–2.14	–2.22
70	1-(2-ethoxyethyl)-1-methylpyrrolidinium bis(trifluoromethylsulfonyl)amide	–2.55	–2.41	–2.27	–2.21	–2.54
71	Ethyl(2-ethoxyethyl)dimethylammonium bis(trifluoromethylsulfonyl)amide	–2.55	–2.35	–2.63	–2.74	–2.58
72	1-(−2-hydroxypropyl)-1-methylpiperidinium chloride	–2.53	–2.27	–2.66	–2.48	–2.50
73	1-cyanomethylpyridinium bis(trifluoromethylsulfonyl)amide	–2.51	–2.74		–2.87	–2.59
74	3-hydroxy-1-undecyloxymethylpyridinium acesultamates	–2.50	–2.62		–2.81	-
75	3-hydroxy-1-undecyloxymethylpyridinium chloride	–2.50	–2.31		–2.27	-
76	1-hexyl-1-methylpyrrolidinium chloride	–2.48	–2.23		–1.84	–2.18
**77**	**1-(2-methoxyethyl)-3-methylimidazolium bis(trifluoromethylsulfonyl)amide**	–2.47	–2.64	–2.51	–2.51	–2.44
78	1-cyanomethylpyridinium chloride	–2.47	–2.75		–2.69	–2.58
**79**	**Ethyl(2-methoxyethyl)dimethylammonium bis(trifluoromethylsulfonyl)amide**	–2.45	–2.55	–2.28	–2.88	–2.57
80	1-(cyanomethyl)-1-methylpiperidinium bis(trifluoromethylsulfonyl)amide	–2.45	–2.83	–2.57	–2.53	–2.54
81	1-(ethoxymethyl)-3-methylimidazolium bis(trifluoromethylsulfonyl)amide	–2.45	–2.48		–2.70	–2.22
82	1-(cyanomethyl)-1-methylpiperidinium chloride	–2.43	–2.84	–2.57	–2.35	–2.53
83	1-butyl-3-methylimidazolium trifluoridotris(pentafluoroethyl)phosphate	–2.40	–2.51		–2.04	
**84**	**3-hydroxy-1-undecyloxymethylpyridinium 1,1-dioxo-1,2-dihydrobenzo[d]isothiazol-3-onate**	–2.40	–2.63		–3.18	
85	1-(2-methoxyethyl)-1-methylpyrrolidinium chloride	–2.38	–2.75	–2.27	–2.42	–2.57
86	1-methyl-1-octylpyrrolidinium chloride	–2.36	–2.07	–2.49	–1.77	–2.01
87	(Ethoxymethyl)ethyldimethylammonium chloride	–2.36	–2.27	–2.30	–2.17	–2.47
**88**	**1-(2-hydroxyethyl)-1-methylpiperidinium bis(trifluoromethylsulfonyl)amide**	–2.34	–2.44	–2.55	–2.62	–2.44
89	Ethyldimethylpropylammonium bis(trifluoromethylsulfonyl)amide	–2.34	–2.01	–2.23	–2.32	–2.12
**90**	**1-(2-hydroxyethyl)-1-methylpiperidinium iodide**	–2.34	–2.44	–2.55	–2.76	–2.44
91	(Ethoxymethyl)ethyldimethylammonium bis(trifluoromethylsulfonyl)amide	–2.30	–2.27	–2.30	–2.35	–2.47
92	Tetrabutylammonium bromide	–2.30	–2.04	–2.19	–2.18	–2.87
93	1-methyl-3-(2-propenyl)imidazolium chloride	–2.30	–2.25		–1.72	–2.44
**94**	**1-methyl-3-propylimidazolium tetrafluoroborate**	–2.29	–2.00	–2.20	–1.70	–2.10
95	1-(2-ethoxyethyl)-3-methylimidazolium bromide	–2.27	–2.22		–2.15	–2.27
96	1-(3-methoxypropyl)-1-methylpiperidinium bis(trifluoromethylsulfonyl)amide	–2.27	–2.59	–2.61	–2.88	–2.43
**97**	**1-methyl-3-propylimidazolium chloride**	–2.27	–2.00	–2.20	–1.78	–2.10
98	1-methyl-3-propylimidazolium hexafluorophosphate	–2.23	–2.00	–2.20	–2.10	–2.19
99	1-ethyl-3-methylimidazolium toluene-4-sulfonate	–2.22	–2.35	–2.06	–2.30	–2.13
**100**	**1-(ethoxymethyl)-1-methylpyrrolidinium bis(trifluoromethylsulfonyl)amide**	–2.22	–2.11		–1.85	–2.43
101	1-propylpyridinium bromide	–2.22	–1.62	–2.25	–1.80	–1.95
**102**	**1-propylpyridinium bis(trifluoromethylsulfonyl)amide**	–2.21	–1.61	–2.25	–1.83	–1.95
103	1-ethyl-3-propylimidazolium bromide	–2.21	–1.85	–1.97	–1.91	–1.84
**104**	**1-(3-methoxypropyl)-1-methylpiperidinium chloride**	–2.20	–2.60	–2.61	–2.70	–2.43
105	1-(ethoxymethyl)-1-methylpiperidinium bis(trifluoromethylsulfonyl)amide	–2.16	–2.30	–2.21	–1.76	–2.10
106	1-hexyl-3-methylimidazolium bis(trifluoromethylsulfonyl)amide	–2.15	–1.87	–1.97	–1.86	–1.91
107	1-butyl-3-methylimidazolium hexafluorophosphate	–2.15	–2.01	–2.02	–2.07	–2.13
108	1-(3-methoxypropyl)pyridinium chloride	–2.15	–2.29	–2.26	–2.11	–2.05
109	1-(ethoxymethyl)pyridinium bis(trifluoromethylsulfonyl)amide	–2.14	–2.05	–2.38	–2.14	–1.98
**110**	**1-(ethoxymethyl)-1-methylpiperidinium chloride**	–2.14	–2.30	–2.21	–1.58	–2.10
111	1-ethyl-3-methylimidazolium hydrogen sulfate	–2.13	–1.92	–2.06	–2.06	–2.13
112	1-butyl-1-methylpyrrolidinium (bis (trifluoromethylsulfonyl)amide)	–2.13	–2.19	–2.00	–2.09	–2.15
113	1-ethyl-3-methylimidazolium trifluoromethanesulfonate	–2.13	–2.04	–2.06	–1.80	–2.13
**114**	**1-heptyl-3-methylimidazolium tetrafluoroborate**	–2.12	–1.79	–2.10	–1.56	–1.81
115	1-(2-ethoxyethyl)-3-methylimidazolium bis(trifluoromethylsulfonyl)amide	–2.12	–2.22		–2.18	–2.28
116	1-ethyl-3-methylimidazolium thiocyanate	–2.12	–1.92	–2.06	–1.96	–2.12
**117**	**1-(2-methoxyethyl)-1-methylpyrrolidinium bis(trifluoromethylsulfonyl)amide**	–2.11	–2.74	–2.27	–2.60	–2.58
118	1-ethylpyridinium chloride	–2.10	–1.70	–1.91	–1.68	–1.97
119	1-(2-methoxyethyl)pyridinium bis(trifluoromethylsulfonyl)amide	–2.09	–2.23	–2.13	–2.45	–2.16
120	1-ethyl-3-methylimidazolium bis(pentafluoroethyl)phosphinate	–2.09	–2.11	–2.06	–2.04	–2.13
**121**	**1-ethyl-3-methylimidazolium bis[1,2-benzenediolato(2-)-O1,O2]borate**	–2.09	–2.61		–2.78	
122	1,1-Dihexylpyrrolidinium tetrafluoroborate	–2.08	–1.81	–1.81	–1.59	–2.05
123	1-ethyl-3-methylimidazolium O-2-(2-methoxyethoxy)ethyl sulfate	–2.08	–2.43	–2.06	–2.07	–2.13
124	1-heptyl-3-methylimidazolium chloride	–2.07	–1.79	–2.10	–1.64	–1.81
125	1-(2-methoxyethyl)pyridinium chloride	–2.07	–2.24	–2.13	–2.27	–2.15
126	1-ethyl-3-methylimidazolium ethyl sulfate	–2.07	–2.00	–2.06	–2.00	–2.13
127	1-ethyl-3-methylimidazolium chloride	–2.06	–1.91	–2.06	–1.80	–2.12
128	1-(3-methoxypropyl)pyridinium bis(trifluoromethylsulfonyl)amide	–2.06	–2.28	–2.26	–2.29	–2.05
129	1-(2-methoxyethyl)-1-methylpiperidinium bromide	–2.06	–2.64	–2.10	–2.15	–2.30
**130**	**Butylethyldimethylammonium chloride**	–2.06	–2.18	–2.23	–2.23	–2.17
**131**	**1-(ethoxymethyl)pyridinium chloride**	–2.06	–2.06	–2.38	–1.97	–1.97
132	1-ethyl-3-methylimidazolium hexafluorophosphate	–2.05	–1.91	–2.06	–2.12	–2.22
133	1-ethyl-3-methylimidazolium tetrafluoroborate	–2.05	–1.91	–2.06	–1.72	–2.13
134	1-benzyl-3-methylimidazolium chloride	–2.05	–2.14		–1.32	–1.71
135	1-ethyl-3-methylimidazolium bis(trifluoromethylsulfonyl)amide	–2.03	–1.90	–2.06	–1.98	–2.13
136	3-methyl-1-octylimidazolium bis(trifluoromethylsulfonyl)amide	–2.03	–1.69	–1.74	–1.80	–1.65
137	Butylethyldimethylammonium bis(trifluoromethylsulfonyl)amide	–2.03	–2.17	–2.23	–2.41	–2.18
138	3-methyl-1-octylimidazolium hexafluorophosphate	–2.03	–1.70	–1.74	–1.95	–1.74
139	1-methyl-1-octylpyrrolidinium Tetrafluoroborate	–2.02	–2.07		–1.69	–2.01
140	1-butyl-3-methylimidazolium iodide	–2.02	–2.01	–2.02	–2.07	–2.04
141	1-butyl-3-ethylimidazolium trifluoromethanesulfonate	–2.01	–1.96	–1.82	–1.73	–1.77
142	1-butyl-3-ethylimidazolium tetrafluoroborate	–2.01	–1.83	–1.82	–1.67	–1.77
**143**	**1-butyl-3-methylimidazolium toluene-4-sulfonate**	–2.00	–2.46	–2.02	–2.24	–2.04
144	1-butyl-3-methylimidazolium thiocyanate	–2.00	–2.02	–2.02	–1.90	–2.04
**145**	**1-ethyl-3-methylimidazolium bis[oxalato(2-)]-borate**	–2.00	–2.14		–2.62	–2.13
146	1-butyl-3-methylimidazolium 1-methanesulfonate	–1.99	–2.16	–2.02	–1.87	–2.04
147	1-butyl-3-methylimidazolium 2-(2-methoxyethoxy)ethyl sulfate	–1.99	–2.54	–2.02	–2.01	–2.04
148	1-butyl-3-methylimidazolium 1-octylsulfate	–1.98	–1.70	–2.02	–2.01	–2.04
**149**	**1-ethyl-3-methylimidazolium tetracyanoborate**	–1.98	–1.93	–2.06	–2.07	–2.13
150	1-butyl-3-methylimidazolium tetrafluoroborate	–1.98	–2.01	–2.02	–1.66	–2.04
151	1-butyl-1-methylpyrrolidinium dicyanamide	–1.98	–2.24	–2.00	–2.01	–2.15
152	1-benzyl-3-methylimidazolium tetrafluoroborate	–1.98	–2.14		–1.23	–1.71
153	1-butyl-3-methylimidazolium hydrogensulfate	–1.97	–2.03	–2.02	–2.01	–2.04
154	1-methyl-3-(2-phenylethyl)imidazolium tetrafluoroborate	–1.97	–1.69		–1.34	–1.77
**155**	**1-hexyl-3-methylimidazolium chloride**	–1.96	–1.88	–1.97	–1.68	–1.91
156	1-butyl-3-methylimidazolium bromide	–1.96	–2.01	–2.02	–1.89	–2.04
157	1-pentyl-3-methylimidazolium tetrafluoroborate	–1.96	–1.96	–1.98	–1.63	–1.97
158	1-butyl-3-methylimidazolium bis(trifluoromethylsulfonyl)imide	–1.96	–2.01	–2.02	–1.92	–2.04
159	1-hexyl-3-methylimidazolium 1,1-dioxo-1,2-benzisothiazol-3(2H)-onate	–1.96	–2.20		–2.58	
**160**	**1-butyl-3-methylimidazolium 1-methylsulfate**	–1.95	–2.17	–2.02	–1.85	–2.04
161	1-butyl-3-methylimidazolium dicyanamide	–1.93	–2.06	–2.02	–1.85	–2.04
162	1-butyl-1-methylpyrrolidinium bromide	–1.93	–2.20	–2.00	–2.05	–2.15
163	1-butyl-3-methylimidazolium trifluoromethanesulfonate	–1.93	–2.14	–2.02	–1.74	–2.04
164	1-(2-methoxyethyl)-1-methylpiperidinium bis(trifluoromethylsulfonyl)amide	–1.93	–2.63	–2.10	–2.19	–2.31
165	1-butyl-1-methylpyrrolidinium chloride	–1.92	–2.20	–2.00	–1.91	–2.15
**166**	**1-heptyl-3-methylimidazolium hexafluorophosphate**	–1.91	–1.79	–2.10	–1.96	–1.90
**167**	**1-butyl-1-methylpyrrolidinium Tetrafluoroborate**	–1.91	–2.20	–2.00	–1.82	–2.15
168	1-butyl-3-methylimidazolium chloride	–1.91	–2.01	–2.02	–1.75	–2.04
169	1-methyl-3-(2-phenylethyl)imidazolium chloride	–1.91	–1.69		–1.42	–1.77
170	1-methyl-3-(2-phenylethyl)imidazolium hexafluorophosphate	–1.90	–1.69		–1.74	–1.86
**171**	**1-hexyl-3-methylimidazolium hexafluorophosphate**	–1.88	–1.88	–1.97	–2.00	–2.00
172	1-hexyl-3-methylimidazolium tetrafluoroborate	–1.88	–1.88	–1.97	–1.60	–1.91
173	1-butylpyridinium trifluoromethanesulfonate	–1.87	–1.77	–1.84	–1.62	–1.83
**174**	**1-methyl-3-[(4-methylphenyl)methyl]imidazolium chloride**	–1.86	–1.70		–1.28	–1.51
**175**	**1-pentyl-3-methylimidazolium hexafluorophosphate**	–1.86	–1.96	–1.98	–2.03	–2.06
176	1-(ethoxymethyl)-1-methylpyrrolidinium chloride	–1.86	–2.12		–1.67	–2.42
177	1-pentyl-3-methylimidazolium chloride	–1.85	–1.96	–1.98	–1.71	–1.97
178	1-hexylpyridinium bis(trifluoromethylsulfonyl)amide	–1.85	–1.61	–1.66	–1.73	–1.54
179	1-hexyl-3-ethylimidazolium tetrafluoroborate	–1.84	–1.70		–1.58	–1.60
180	1-hexylpyridinium trifluoromethanesulfonate	–1.84	–1.75	–1.66	–1.55	–1.54
181	1-butylpyridinium hexafluorophosphate	–1.84	–1.64	–1.84	–1.94	–1.92
**182**	**1-butyl-1-methylpiperidinium bromide**	–1.83	–2.12	–1.90	–1.98	–1.82
**183**	**1-butyl-3-methylimidazolium hexafluoroantimonate**	–1.81	–2.04		–1.92	
184	1-butylpyridinium tetrafluoroborate	–1.80	–1.64	–1.84	–1.54	–1.83
185	1-butyl-1-methylpiperidinium bis(trifluoromethylsulfonyl)amide	–1.78	–2.11	–1.90	–2.01	–1.82
186	1-butylpyridinium bromide	–1.77	–1.64	–1.84	–1.76	–1.83
187	1-hexyl-3-ethylimidazolium bromide	–1.77	–1.70		–1.81	–1.60
**188**	**1-hexylpyridinium hexafluorophosphate**	–1.76	–1.62	–1.66	–1.88	–1.63
**189**	**1-butylpyridinium O-methyl sulfate**	–1.75	–1.80	–1.84	–1.72	–1.83
190	1-benzyl-3-methylimidazolium hexafluorophosphate	–1.74	–2.14		–1.64	–1.80
191	1-hexylpyridinium chloride	–1.72	–1.62	–1.66	–1.55	–1.54
**192**	**1-butylpyridinium chloride**	–1.70	–1.64	–1.84	–1.62	–1.83
**193**	**1-decyl-3-methylimidazolium hexafluorophosphate**	–1.68	–1.52		–1.87	–1.32
194	1-butyl-4-methylpyridinium trifluoridotris(pentafluoroethyl)phosphate	–1.64	–1.96	–1.56	–1.85	–1.55
195	3-methyl-1-nonylimidazolium hexafluorophosphate	–1.62	–1.54	–1.44	–1.90	–1.54
196	3-methyl-1-octylimidazolium chloride	–1.60	–1.70	–1.74	–1.62	–1.65
197	1-octylpyridinium chloride	–1.60	–1.48	–1.64	–1.50	–1.25
198	1-butyl-3-methylimidazolium bis(trifluoromethyl)amide	–1.60	–2.10		–1.87	–2.04
199	1-pentylpyridinium bis(trifluoromethylsulfonyl)amide	–1.55	–1.63	–1.56	–1.77	–1.67
200	1-Butyl-4-methylpyridinium Tetrafluoroborate	–1.54	–1.45	–1.56	–1.48	–1.52
201	3-methyl-1-octylimidazolium tetrafluoroborate	–1.53	–1.70	–1.74	–1.54	–1.65
**202**	**1-pentylpyridinium bromide**	–1.52	–1.64	–1.56	–1.74	–1.66
**203**	**1-Hexyl-4-methylpyridinium Tetrafluoroborate**	–1.48	–1.34	–1.51	–1.44	–1.38
204	1-butyl-4-methylpyridinium tetracyanidoboranuide	–1.46	–1.47	–1.56	–1.83	–1.52
**205**	**1-hexyl-4-methylpyridinium chloride**	–1.44	–1.34	–1.51	–1.53	–1.38
206	1-butyl-4-methylpyridinium chloride	–1.44	–1.45	–1.56	–1.56	–1.52
**207**	**3-methyl-1-nonylimidazolium tetrafluoroborate**	–1.43	–1.54	–1.44	–1.50	–1.45
208	1-butyl-4-methylpyridinium hexafluorophosphate	–1.43	–1.45	–1.56	–1.88	–1.61
**209**	**1-octylpyridinium bis(trifluoromethylsulfonyl)amide**	–1.40	–1.47	–1.64	–1.68	–1.26
210	1-methyl-3-octylimidazolium O-octyl sulfate	–1.38	–1.39		–1.89	
211	3-methyl-1-nonylimidazolium chloride	–1.36	–1.54	–1.44	–1.58	–1.45
212	1-Butyl-3-methylpyridinium Tetrafluoroborate	–1.27	–1.49		–1.48	–1.28
213	3-hexyl-1,2-dimethylimidazolium tetrafluoroborate	–1.27	–1.65		–1.44	–1.17
214	1-butyl-3-methylpyridinium hexafluorophosphate	–1.24	–1.49		–1.89	–1.37
215	1-butyl-3-methylpyridinium dicyanidoamide	–1.22	–1.54		–1.67	–1.28
216	1-octyl-4-methylpyridinium Tetrafluoroborate	–1.22	–1.16		–1.35	–1.12
217	1-butyl-3,5-dimethylpyridinium Tetrafluoroborate	–1.17	–1.25		–1.44	–1.10
218	1-butyl-3-methylpyridinium chloride	–1.15	–1.49		–1.57	–1.28
219	1-octyl-4-methylpyridinium chloride	–1.11	–1.16	–1.11	–1.44	–1.12
**220**	**1-butyl-3,4-dimethylpyridinium Tetrafluoroborate**	–1.10	–1.06	–1.30	–1.39	–1.12
221	1-decyl-3-methylimidazolium chloride	–1.09	–1.52	–1.13	–1.54	–1.23
222	1-decyl-3-methylimidazolium tetrafluoroborate	–1.08	–1.52	–1.13	–1.46	–1.23
223	1-hexyl-3-methylpyridinium chloride	–1.06	–1.53		–1.51	–1.07
**224**	**1-butyl-3,5-dimethylpyridinium chloride**	–0.99	–1.25		–1.52	–1.10
225	4-(dimethylamino)-1-ethylpyridinium bromide	–0.99	–0.64	–0.95	–1.59	–1.08
226	3-methyl-1-octadecylimidazolium chloride	–0.96	–0.62	–0.97	–1.30	–1.12
227	4-(dimethylamino)-1-ethylpyridinium bis(trifluoromethylsulfonyl)amide	–0.93	–0.63	–0.95	–1.63	–1.08
228	1-decyl-3-ethylimidazolium bromide	–0.92	–1.45		–1.18	–0.87
**229**	**1-decyl-3-methylimidazolium bromide**	–0.92	–1.52		–1.69	
230	1-butyl-3,4-dimethylpyridinium chloride	–0.85	–1.06		–1.48	–1.12
231	1-butyl-2-methylpyridinium Tetrafluoroborate	–0.82	–1.27		–1.47	–1.15
**232**	**4-(dimethylamino)-1-hexylpyridinium bis(trifluoromethylsulfonyl)amide**	–0.81	–0.66	–0.63	–1.51	–0.68
**233**	**1-butylquinolinium bromide**	–0.79	–1.15		–1.06	–0.77
**234**	**Benzyldecyldimethylammonium chloride**	–0.73	–2.46		–1.48	–1.22
235	1-butyl-2-methylpyridinium chloride	–0.70	–1.27		–1.55	–1.15
236	1-hexadecyl-3-methylimidazolium chloride	–0.68	–0.83		–1.38	–0.50
**237**	**1-octyl-3-methylpyridinium chloride**	–0.64	–1.31		–1.42	–0.79
238	1-butylquinolinium tetrafluoroborate	–0.62	–1.15		–0.83	–0.78
239	4-(dimethylamino)-1-butylpyridinium Chloride	–0.60	–0.68	–0.63	–1.39	–0.91
**240**	**3-methyl-1-tetradecylimidazolium chloride**	–0.54	–1.12		–1.41	–0.47
241	4-(dimethylamino)-1-hexylpyridinium chloride	–0.50	–0.67		–1.33	–0.67
242	1-hexylquinolinium tetrafluoroborate	–0.48	–0.93		–0.76	–0.78
243	1-octylquinolinium tetrafluoroborate	–0.30	–0.89	–0.83	–0.69	–0.26
						

a Bold ILs refer to the test set.

### 2.3 Previously Developed Linear and Nonlinear QSAR Models

In addition to the incorporation of novel quantum descriptors (COSMO-RS descriptors), the primary aim of this work is to develop a predictive model with improved accuracy. Furthermore, the outcomes of this model are systematically compared with those reported in previous studies to assess its relative performance. As illustrated in Table 1, various linear and nonlinear models have been proposed in the literature. However, many of these models suffer from limitations such as reliance on a small set of ILs,^7,8^ insufficient predictive power,^10^ or failure to account for the influence of anionic structural features on toxicity predictions.^5,8,11,13^ To enable a meaningful comparison, several statistical parameters like the coefficient of determination (*R* ^2^) have been extracted from previous works and are reported alongside the results of the present model. By providing a transparent and quantitative comparison with existing models, this study not only highlights the strengths of the proposed approach but also aims to guide future developments in the field of toxicity prediction for ILs.

### 2.4 Data-Driven QSAR Approach

#### 2.4.1 Calculation of COSMO-Based Molecular Descriptors

The molecular descriptors utilized in this study are derived from σ-profiles, computed using the COSMO-RS methodology.^22–25^ These σ-profiles were obtained from the COSMObase 2023 database, integrated within the COSMOtherm 2023 software suite,^26^ and were generated using the TZVP basis set. All descriptor values correspond to the lowest-energy conformations of the respective cationic and anionic species. In essence, the σ-profile offers a two-dimensional representation of the molecular surface polarity in three dimensions, as depicted in Figure 1. The horizontal axis indicates the magnitude of the surface charge density (SCD), while the vertical axis reflects the likelihood (or frequency) of encountering a particular SCD across the molecular surface. These profiles are computed at intervals of 0.001, typically spanning a range from −0.03 to +0.03 e/Å^2^. An example of sigma profile descriptors for cation and anion is shown in Figure 1.

**Figure 1:**
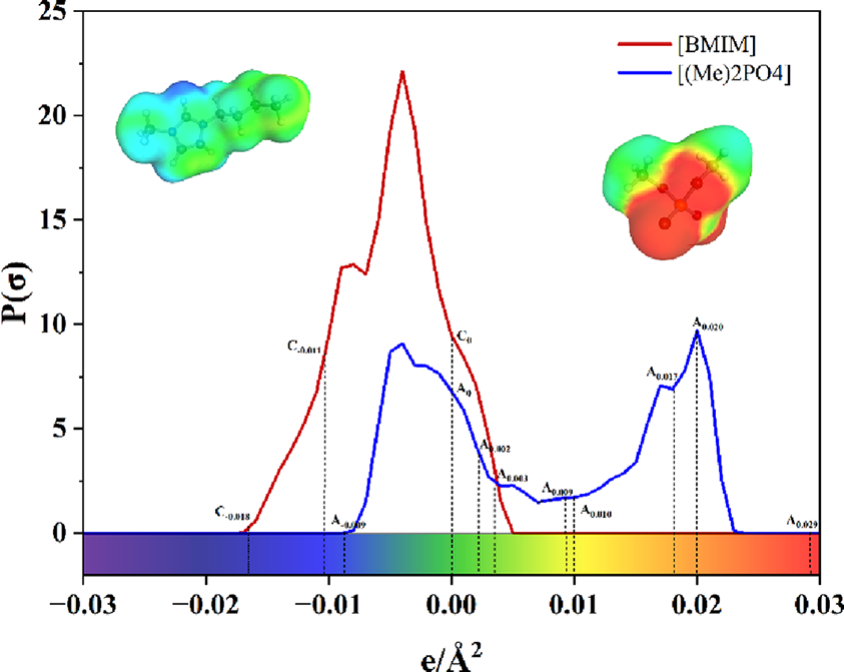
Demonstration of some cationic and anionic descriptors (i.e., values of probability (amount) at some surface charge densities (SCD)) for [BMIM] and [(Me)_2_PO_4_]).

#### 2.4.2 Model Development

Equation 1 illustrates that Log 1/EC_50_ depends on both cation and anion descriptors. To construct an accurate model, it is crucial to identify and select the most relevant descriptors from the available 122 sigma profile variables, a step that was notably lacking in a previous study by Cho and Yun.^10^ Several established techniques exist for variable selection, including the genetic algorithm (GA),^27^ artificial neural networks (ANN),^28^ and the replacement method (RM).^29^ In this research, GA was employed to develop multiple linear regression (MLR) QSAR models based on the COSMO descriptors. Further information on the GA-MLR approach can be found in refs 30,31 . According to the QSARINS modeling workflow, descriptor selection via a GA was performed solely on the training set, and the test set was employed only to evaluate the predictive ability of the final model.

#### 2.4.3 Statistical Parameters

To ensure the reliability of the QSAR model, it is essential to evaluate its performance by using established statistical indicators. These include the determination coefficient (*R* ^2^), the cross-validated determination coefficient from leave-one-out analysis (*Q* ^2^_LOO–CV), the adjusted *R* ^2^ (*R* ^2^_adj), the percentage of average absolute relative deviation (%AARD), the average absolute deviation (AAD), the Fisher statistic (*F*-value), the root mean squared error (RMSE), the standard deviation of residuals (*S*), and the maximum allowable leverage (h*). Further explanations and the corresponding equations for these metrics are provided in Table 3 (see eqs 2–10).

**Table 3:** Applied Statistical Parameters in This Study^a^

introduced parameters	introduced parameters equations	eqs no
coefficient of determination	$R^{2}=1-\frac{\sum_{i=1}^{n}(Y_{i}^{\exp}-Y_{i}^{\mathrm{pre}}{)}^{2}}{\sum_{i=1}^{n}(Y_{i}^{\exp}-{\overset{-}{Y}}_{i}{)}^{2}}$	(2)
adjustable coefficient of determination	${R^{2}}_{\mathrm{Adj}}=1-(1-R^{2})\times \left(\frac{n-1}{n-p-1}\right)$	(3)
leave-one-out cross-validated coefficient of determination	${Q^{2}}_{\mathrm{LOO}-\mathrm{CV}}=1-\frac{\sum_{i=1}^{n}(Y_{i}^{\exp}-Y_{i}^{\mathrm{pre}-\mathrm{CV}}{)}^{2}}{\sum_{i=1}^{n}(Y_{i}^{\exp}-Y{®}_{i}{)}^{2}}$	(4)
Fisher	F=∑i=1n(Yipre−Y−i)2p∑i=1n(Yiexp−Yipre)2n−p−1	(5)
standard residual	$S=\sqrt{\frac{\sum_{i=1}^{n}(Y_{i}^{\exp}-Y_{i}^{\mathrm{pre}}{)}^{2}}{n-p-1}}$	(6)
root mean squared error (RMSE)	$\mathrm{RMSE}=\sqrt{\frac{\sum_{i=1}^{n}(Y_{i}^{\exp}-Y_{i}^{\mathrm{pre}}{)}^{2}}{n}}$	(7)
average absolute deviation	$\mathrm{AAD}=\frac{\sum_{i=1}^{n}(|Y_{i}^{\exp}-Y_{i}^{\mathrm{pre}}|)}{n}$	(8)
average absolute relative deviation %	$\mathrm{AARD}\%=\frac{\sum_{i=1}^{n}(|Y_{i}^{\exp}-Y_{i}^{\mathrm{pre}}|)/Y_{i}^{\exp}}{n}\times 100$	(9)
maximum leverage	h* = 3(*p* + 1)/*n*	(10)

a *Y_i_* ^exp^, *Y_i_* ^pre^, ${\overset{-}{Y}}_{i}$ , *n*, and *p* demonstrate experimental values, predicted values, average experimental values, the number of the experimental dataset, and the number of employed descriptors, respectively.

Applicability Domain (AD) analysis, as a vital concept of the QSAR approach, is considered. It allows:^32^ (1) the uncertainty in prediction, (2) the extent of extrapolation of QSAR models.^33,34^ In order to predict Log 1/EC50 for a new IL, it is essential that a new IL lies within the same AD space. It means that new IL is physicochemically, biologically, or structurally similar to molecules used for model development (i.e., training set). The larger the space of AD, the more reliable are the predictions of new ILs. To carry out the external validation using a validation set, it is essential to ensure that the validation set of molecules is inside the QSAR model’s AD.^35^

The space of AD can be specified using two main parameters: (1) the leverage values (*h_i_*) and (2) the standardized residual (SDR). SDR was defined as eq 11:

$$\mathrm{SDR}=\frac{Y_{i}^{\exp}-Y_{i}^{\mathrm{pre}}}{\sqrt{\frac{\sum_{m=1}^{n}(Y_{i}^{\exp}-Y_{i}^{\mathrm{pre}}{)}^{2}}{n}}}$$ 11

*h_i_*, represents a measure of a molecule’s distance from the center of the training set. It is needed to determine whether new ILs are within the AD of the developed QSAR model or not. The parameter can be calculated with eq 12.

$$h_{i}\;(\mathrm{or}\;\mathrm{Leverage}(i))=z_{i}\cdot ({Z_{i}}^{T}Z_{i}{)}^{-1}\cdot {z_{i}}^{T}$$ 12

when *z_i_*, *Z* is the descriptor row vector of point *i* and a *n* × *p* matrix of descriptors for compounds derived from the training set, respectively. AD of developed QSAR models can be obtained in QSARINS software for each model, and maximum leverage (i.e., h*) can be calculated using eq 10.

#### 2.4.4 External and Internal Validations

Once the QSAR model is constructed, it is crucial to perform both internal and external validations to assess its robustness and predictive accuracy. The training set, comprising approximately 75% of the full data set, is used for internal validation, while the remaining ∼25% is reserved for external validation. For external assessment, the predictive strength of the model was tested using a random exclusion method, where selected data points were arbitrarily assigned to the validation set. For internal validation, the model was subjected to several diagnostic procedures, including Y-randomization, leave-multiple-out cross-validation (LMO–CV), and leave-one-out cross-validation (LOO–CV). These internal checks were exclusively carried out on the training set to ensure the model’s reliability and to detect potential overfitting or chance correlations.

## 3 Results

In this study, the primary data set comprising 243 ILs was randomly divided into training and test sets with a 75:25 ratio (i.e., 183 ILs in training and 60 ILs in test). This split was implemented to enable both internal and external evaluations using a defined and meaningful set of statistical parameters. The ILs included in the test set are highlighted in bold in Table 2. Before presenting the main findings, it is important to determine the optimal number of molecular variables (i.e., molecular COSMO-RS descriptors) for developing the MLR model for this data set. This was assessed using a breaking plot analysis, as shown in the Supporting Information Excel file (Sheet 2). The analysis revealed that a model using 11 variables achieves nearly the same predictive performance as models with more variables. For instance, the 11-variable model had an *R* ^2^ of 0.82, compared to 0.83 for a 12-variable model. This suggests that including additional variables beyond the optimal 11 offers negligible improvement in accuracy and is therefore unnecessary. Despite this, Wu et al.^5^, Peric et al.,^12^ Yan et al.,^15^ and Torrecilla et al.^16^ used 14, 10, 17, and 12 input variables to develop nonlinear or linear models for those data sets, including just 160, 55, 221, and 153 data points, respectively (see Table 1). Hence, for those data sets, it is strongly recommended to limit the input to fewer descriptors, and for using additional descriptors, a rational justification and interpretation are required. Therefore, a mismatch was frequently observed between the number of descriptors used and the structural diversity of the data sets, which could result in overfitting or produce misleading performance indicators. On the contrary, and as shown in Table 1, the study by Cho and Yun,^10^ which investigated the largest number of ILs (251 data points) and developed a model using a limited number of descriptors, based on their own unique training and test sets. Owing to this, the statistical performance of their model was expectedly low, with *R* ^2^ values around 0.74. Initially, we needed to evaluate whether these six descriptors could adequately predict our studied data set (our training (183 ILs) and test (60 ILs) sets). As illustrated in Figure 2, these descriptors fail to provide satisfactory predictions for both the training and test sets. As the statistical parameters obtained for our training and test sets are close to those reported values (i.e., R2-Train = 0.74 and R2-Test = 0.71) in Cho and Yun,^10^ it is evident that these descriptors are insufficient to improve model accuracy.

**Figure 2:**
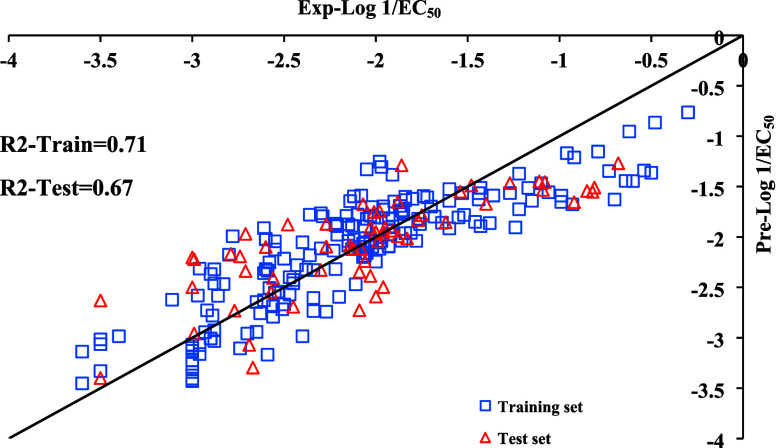
Predicted versus experimental values (Log 1/EC_50_) for both of training (square) and test (triangle) sets using new developed MLR model including proposed descriptors by Cho and Yun.^10^

Therefore, a new predictive model including new descriptors is required to enhance the predictive performance. A new model incorporating quantum chemical descriptors is presented in Table 4. A more comprehensive comparison is provided in Table 2, which includes predicted values from both our model and the one proposed by Cho and Yun.^10^ According to the shown prediction by a model including Cho and Yun’s descriptors (see Figure 2)) and the former predicted values proposed by Cho and Yun^10^ (see Table 2), it has been clearly demonstrated that the new model-based COSMO-RS descriptors in this work have a superior accuracy in the prediction of IL’s toxicity.

**Table 4:** Suggested MLR-QSAR Model for the Training Set Including 183 Data Points

type of structural variable	number of data points (or ILs) in training set	model	eq no
COSMO-RS	183	Log 1/EC_50_= 0.0557 **C-0.008** – 0.1161 **C-0.004** + 0.1529 **C-0.003** + 0.1482 **C0.002** – 0.3281 **C0.003** + 0.1583 **C0.005** – 0.3812 **C0.009** – 0.3416 **C0.014** – 0.0536 **A-0.006** + 0.0159 **A-0.001** – 0.0114 **A0.004** – 2.6595	(13)

As can be seen in eq 13, the developed model has both cationic and anionic descriptors, which means both effects have been considered in the prediction. Despite former models^5,8,11,13^ which just included cationic descriptors and without any anionic descriptors, it should be added that the presence of some anions alongside a constant cation can change the toxicity values, noticeably. Therefore, both cationic and anionic descriptors should be present in the developed predictive model. For instance, ([1-butyl-3-methylimidazolium] [trifluoridotris­(pentafluoroethyl)­phosphate] (i.e., IL-83) and [1-butyl-3-methylimidazolium] [bis­(trifluoromethyl)­amide] (i.e., IL-198)) and ([Tetrabutylphosphonium] [bis­[1,2-benzenediolato­(2-)-O1,O2]­borate] (i.e., IL-9) and [Tetrabutylphosphonium] [bromide] (i.e., IL-56)) have constant cations and two different anions. However, the measured toxicity values for both pairs have significant differences (see Table 2). Such other pairs can be frequently found in our studied data set which expresses the unavoidable effect of anion’s structures on the toxicity values. However, this effect is much lower than the cation effect. These points decline some results by Wu et al.^5^ that only cationic components play a dominant role in influencing the toxicity of ILs toward the AChE enzyme. Moreover, a total of 24 data points were removed from the data set based on the identification of activity cliffs structurally similar ILs exhibiting markedly different biological activities in Wu et al.^5^ study. While this strategy was employed to improve the overall statistical performance and predictive accuracy of the model by reducing noise, it may also introduce a form of bias. Excluding such structurally informative outliers could limit the model’s ability to capture the full complexity and nonlinearity inherent in structure–toxicity relationships. In contrast, the present study retains these challenging data points in the modeling process, aiming to develop a more robust and realistic model that reflects the true diversity of IL’s behaviors. This approach may lead to slightly lower statistical metrics but offers greater generalizability and reliability when applied to structurally diverse or novel compounds. It seemed that a more detailed analysis was still necessary to identify the specific molecular features that most significantly impact AChE activity. As can be seen in eq 13, the number of cationic descriptors appearing is more than that of anionic descriptors, which emphasizes the noticeable effect of cations on the toxicity values. It should be added that sometimes the effect of cations on the toxicity of ILs is so pronounced that simply replacing an alkyl substituent within the cationic ring can lead to a significant change in the toxicity of the IL. For instance, 1-octyl-3-methylpyridinium chloride and 1-octyl-4-methylpyridinium chloride, which differ only in their cationic substituents while sharing the same anion, exhibit markedly different measured toxicity values (see Table 2). In fact, this cationic influence appears to be even more significant than the effect of anions discussed earlier. It appears that, in some studies involving fewer than 200 structures of ILs (see Table 1), the selected data sets were possibly chosen in such a way that the influence of anions was minimal. As a result, anionic descriptors were entirely absent from the final models. This observation stands in contrast with other findings, where the impact of certain anions is shown to be substantial. The present study aims to address and resolve these inconsistencies. All in all, it was expected that the developed model would have a greater number of cationic descriptors in comparison to anionic descriptors. The values of statistical parameters for eq 13 are presented in Table 5.

**Table 5:** Values of Statistical Parameters of the Suggested MLR-QSAR Models for Both Training and Test Sets

eqs. no	sets	number of data points	*R* ^2^	*R* ^2^-Adj	*Q* ^2^-LOO	*Q* ^2^-LMO	*F*	*S*	RMSE	AAD	%AARD
(13)	training	183	0.821	0.810	0.789	0.785	71.49	0.2908	0.2811	0.222	13.5
	test	60	0.751						0.3500	0.234	17.6

To further assess the robustness and stability of the developed linear QSAR model, extensive internal validation was carried out. Specifically, 1000 random subsamplings were performed in which approximately 20% of the training data were repeatedly excluded for validation. The obtained *Q* ^2^-LMO values consistently averaged around 0.78, clearly indicating that the model is not only statistically significant but also highly stable across different random partitions of the training set. Such consistent predictive performance demonstrates the reliability of the selected molecular descriptors and confirms that the observed correlations are not numerical artifacts arising from the fitting process. As shown in Table 5, the MLR-QSAR model developed using COSMO-RS descriptors demonstrated sufficiently high *Q* ^2^_LOO_ and *Q* ^2^_LMO_ values (internal validation), confirming its reliable predictive performance for the prediction of Log 1/EC_50_ against AChE of ILs. To further evaluate model robustness, Y-scrambling (*R* ^2^-Yscr = 0.06) was performed on the training set using the QSARINS software. The outcomes of these tests supported the validity of the model (see the Supporting Information Excel file (Sheet 3)). To comprehensively compare with the former QSTR-model by Kumar and Kumar,^36^ extra statistical criteria such as concordance correlation coefficient (CCC), *Q* ^2^_F1_, *Q* ^2^_F2_, *Q* ^2^_F3_, and novel metrics (r2m-average and r2m-delta) as well as *R* ^2^_Y‑scrambling_, *Q* ^2^-_Y‑scrambling_, and *R* ^2^_Y‑randomization_ have been reported in Table 6.

**Table 6:** Values of Concordance Correlation Coefficient (CCC), Q2F1, Q2F2, Q2F3, and Novel Metrics (r2m-avaerage and R2m-delta) As Well As R2Y-scrambling, Q2-Y-scrambling, and R2Y-randomization

eqs no	sets	number of data points	CCC	Q2F1	Q2F2	Q2F3	R2m-Aver	R2m-delta	R2-Yscr	Q2-Yscr	R2-Yran
(13)	training	183	0.902						0.060	–0.078	0.059
	test	60	0.853	0.734	0.729	0.724	0.648	0.205			

Regarding the external validation, the model achieved an *R* ^2^ of approximately 0.75 for the independent test set, which can be considered a satisfactory predictive performance for toxicity end points of ILs. Importantly, this level of predictivity is in line with previously published studies,^36,37^ including the work by Miao et al.,^37^ where even more complex machine learning approaches yielded comparable test-set correlations. In fact, the authors of that study^37^ explicitly acknowledged the difficulty of accurately predicting AChE toxicity in the test set. Thus, achieving a 0.75 correlation with a linear model further highlights the strength and generalizability of the present approach. All in all, our present model accurately predicted the Log 1/EC_50_ against AChE of ILs in the test set, as indicated by low RMSE = 0.35 and %AARD = 17.6 values. The proposed MLR-QSAR model (eq 13) effectively captured the influence of cation and anion structures on Log 1/EC_50_ against AChE for most of the ILs in the data set. The Williams plot for both training and test sets, generated using eq 13, is presented in the Supporting Information Excel file (Sheet 4). This plot reveals that the data set contains a single outlier from the test set, characterized by a leverage value exceeding the critical threshold (h* = 0.197) and standardized residuals slightly outside the ±3 range. Although some ILs exhibited leverage values above the threshold, the model still provided accurate predictions for their toxicity. Finally, the plots comparing predicted versus experimental values for both the training and test sets, obtained using eq 13, are illustrated in Figure 3.

**Figure 3:**
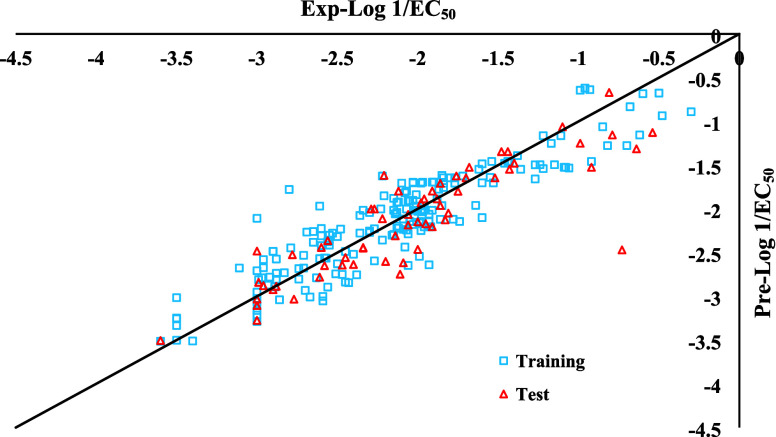
Predicted versus experimental values (Log 1/EC_50_) for both of training and test sets using eq 13.

Equation 13 utilizes eight sigma profile charge density points (‘C-0.008’, ‘C-0.004’, ‘C-0.003’, ‘C0.002’, ‘C0.003’, ‘C0.005’, ‘C0.009’, ‘C0.014’) as cationic descriptors, while three points (‘A-0.006’, ‘A-0.001’, and ‘A0.004’) as anionic descriptors. The probabilities associated with these charge density points for each cation and anion are used to predict the Log 1/EC_50_ against AChE of ILs. With the presence of such a model that incorporates both cationic and anionic quantum descriptors, it becomes relatively straightforward to identify which ILs are more toxic and which are less toxic. In other words, the higher the value of the parameter Log 1/EC_50_, the lower the EC_50_, which in turn indicates a higher toxicity of the corresponding IL. The values of sigma profile charge density descriptors with the aim of predicting the toxicity of new ILs with these studied cations and anions have been reported in the Supporting Information Excel file (Sheet 5). The main advantage of this study is that Log 1/EC_50_ against AChE for those ILs whose values have not been reported in the literature yet can be calculated using eq 13 with respect to AD.

In QSAR modeling, it is well established that, particularly for classical QSAR/QSPR models based on MLR, the descriptors included in the final model should be as mutually independent as possible. This is important because high intercorrelation among descriptors can lead to overfitting, instability of model coefficients, and loss of interpretability. In the present study, although two out of the 11 selected descriptors show some degree of correlation, the majority of the descriptors are statistically independent of each other. Particular attention was paid during descriptor selection to ensure minimal redundancy, in order to enhance the model’s robustness and generalizability. It is noteworthy that this critical aspect of descriptor independence was not referred to in many of the related studies reviewed in Table 1. This omission represents yet another gap in earlier models developed for predicting the toxicity of ILs. By addressing this issue, the current work aims to provide a more reliable and scientifically sound foundation for future QSAR investigations in this area. The intercorrelation between the used descriptors of this study and even those descriptors which had already been used in Cho and Yun study^10^ and Miao et al.^37^ have been shown in the Supporting Information Excel file (Sheet 6). It is worth noting that certain descriptor intercorrelations were observed in the final models.^10,37^ Such phenomena are not uncommon in QSTR/QSAR studies, particularly when descriptors describe related structural or physicochemical features. For example, in the study,^37^ a strong correlation was reported between fr_Ar_N and fr_aryl_methyl (*r* = 0.84), reflecting their shared representation of molecular polarity. In practice, descriptor intercorrelation may arise naturally due to overlapping chemical information, and it is often difficult to completely avoid. Importantly, many previously reported models, including those summarized in Table 1, did not explicitly address this issue. Therefore, the presence of some intercorrelated descriptors in the present work does not undermine the validity of the model but rather reflects the inherent complexity of chemical structure–toxicity relationships. Only in two former studies conducted by Cho and Yun,^10,37^ the interrelationships between descriptors were calculated. The correlation values between the descriptors used in this study and those used previously^10,37^ demonstrate that the selected descriptors are largely independent. Although it has previously been noted (see Figure 2)), that the descriptors proposed by Cho and Yun^10^ may not have demonstrated sufficient predictive power for the prediction of the toxicity of ILs, the present analysis reveals that those descriptors exhibit a level of mutual independence comparable to that observed among the quantum-based descriptors (i.e., COSMO-RS) used in this study. Such points highlight the importance of evaluating descriptor independence as a fundamental criterion in QSAR model development, regardless of the final model’s overall accuracy. It would have been beneficial if such descriptor correlation analyses had been transparently reported in all of the studies listed in Table 1, as this would have provided a clearer assessment of the descriptor redundancy and model quality.

On the other hand, as shown in Table 2 (or the Supporting Information Excel file (Sheet 1)), among the 29 experimentally studied anions in combination with various cations, some anions such as bis­(trifluoromethylsulfonyl)­amide, chloride, and tetrafluoroborate have been extensively investigated, while others such as 1-methanesulfonate and 4-methylbenzenesulfonate have been reported only once and in combination with a single cation. This observation indicates that the lack of a significant effect on the experimental toxicity by certain anions in the presence of specific cations does not necessarily imply a general rule for all anions. Therefore, further experimental and computational studies are still required to develop a more comprehensive understanding of anion effects. But this study clearly demonstrated that neglecting the effects of anions and relying solely on cationic descriptors results in statistically and logically poor models. Such models tend to predict nearly identical toxicity values for all ILs that share the same cation but differ in their anions, which is clearly inconsistent with experimental observations. These discrepancies are evident in many previous studies as well.^5,8,11,13^ As shown in Table 2, two earlier linear QSAR models, such as those developed by^8,15^ failed to effectively capture the influence of anions on the toxicity of ionic liquids. This limitation significantly undermined their predictive reliability. In contrast, the model proposed in this study addresses this critical shortcoming, offering a more comprehensive representation of both the cationic and anionic contributions. Consequently, the earlier models are not competitive with our approach in terms of accuracy. Among the previous studies listed in Table 1, it could be argued that both nonlinear models (i.e., SVM and CCN) proposed by Basant et al.,^11^ stands out due to their use of a reasonably sized data set (i.e., 232 ILs) and their overall strong predictive performance (i.e., *R* ^2^ = 0.97). However, it appears that these models also excluded data points in which the effect of anions on IL’s toxicity was substantial. The final data set used in that study included 232 ILs, most of which showed minimal variation in toxicity due to differences in anions in the presence of constant cations. Consequently, the final models of both the SVM and CCN approaches tended to predict nearly identical toxicity values for ILs sharing the same cation but differing in their anions. This inconsistency is not observed in our present study, where the effect of anions is clearly reflected in the model’s predictions. In fact, the only previous model that explicitly considered the influence of anions using a large and diverse data set of ILs (i.e., 251) was the one proposed by Cho and Yun^10^ (see Tables 1 and 2), although its final model lacked sufficient predictive accuracy. In another study conducted by Yan et al.,^15^ an attempt was made to consider the effect of anions, and a predictive model was developed based on a data set comprising 221 ILs. The model employed a total of 17 molecular descriptors, of which 16 were cation-based, and only one represented an anionic descriptor. This limited inclusion of anion-specific descriptors appears insufficient to adequately capture the influence of anions on the toxicity of the ILs. As a result, the model may not fully reflect the complex interplay between the cationic and anionic components in determining overall toxicity. These observations motivated us to develop a new, more comprehensive, and yet simpler QSAR model in the present work, capable of more accurately predicting the toxicity of ILs across a broader chemical space.

To further validate the proposed QSAR model, we compared our data set of 243 ILs with the 229 ILs studied by Miao et al.^37^ and Kumar and Kumar.^36^ Thirteen ILs from their data sets were absent in our training/test sets (for details, see Supporting Information Excel file (Sheet 7)). Among these, descriptor values for both the cation and the anion were available for only two ILs. These two ILs were used as independent test cases to demonstrate the predictive capability of the model (i.e., eq 13). The results have been shown in Table 7.

**Table 7:** Experimental and Predicted Values (Using eq 13), along with the Corresponding Descriptor Values (Using Sheet 5), for the Two New ILs That Were Not Included in the Training or Test Sets of This Study

two new ILs	**Exp**	C-0.008	C-0.004	C-0.003	C0.002	C0.003	C0.005	C0.009	C0.014	A-0.006	A-0.001	A0.004	**Pre**
1-(ethoxymethyl)-3-methylimidazolium chloride	–2.61	13.681	15.909	11.369	4.648	1.598	0.548	1.053	0.987	0	0	0	–2.49
4-(dimethylamino)-1-butylpyridinium bis(trifluoromethylsulfonyl)amide	**–0.59**	14.142	24.318	29.208	8.783	5.369	0.038	0	0	0.438	7.713	8.108	**–0.67**

This ensures that validation is performed on entirely new ILs, not included in the original training or test sets (but both cation and anion included in Sheet 1), and confirms the applicability of the QSAR model for new ILs where descriptors are available in the Supporting Information Excel-file (Sheet 5).

It should be noted that the validation strategy adopted in this study is based on random data splitting and conventional cross-validation protocols, which may lead to optimistic estimates of the predictive performance for ILs. As clearly demonstrated by Makarov et al.,^38^ ILs are equimolar binary systems, and rigorous evaluation of model generalization requires component-based validation schemes in which identical cations or anions are excluded from both training and test sets. Owing to software limitations, such strict validation could not be implemented in the present work; therefore, the reported prediction errors mainly reflect interpolation within the studied chemical space, while prediction errors for truly novel ILs containing unseen ions are expected to be significantly larger.

Recent advances in ML have demonstrated that representation-learning approaches, including models based on molecular graphs and natural language–style representations such as SMILES, can achieve high predictive accuracy for toxicological end points, as evidenced by the outcomes of the Tox24 Challenge.^39^ In particular, fine-tuned foundation models and multitask learning frameworks have shown a strong performance relative to traditional descriptor-based QSAR models. While the present study employs an interpretable MLR framework with COSMO-RS descriptors to provide mechanistic insight into IL toxicity, emerging AI and explainable AI (XAI) methods offer promising complementary tools for mixture modeling and complex structure–toxicity relationships. Future work may explore hybrid strategies that integrate physically meaningful quantum-chemical descriptors with modern representation-learning approaches to further enhance predictive accuracy while retaining interpretability for regulatory and risk assessment applications.

## 4 Conclusion

This study presents a novel MLR-QSAR model developed using COSMO-RS quantum descriptors for predicting the toxicity (Log 1/EC50) of ILs toward the acetylcholinesterase (AChE) enzyme. By analyzing a data set of 243 ILs, it was demonstrated that an optimal set of 11 descriptors was sufficient to achieve high predictive accuracy, thereby avoiding unnecessary model complexity. Unlike earlier models that often relied solely on cationic descriptors, this model includes both cationic and anionic contributions, allowing for a more realistic representation of IL’s toxicity. While cations were found to exert a stronger influence overall, several examples illustrated the significant impact of specific anions, especially when paired with a constant cation. The model was rigorously validated using internal (*Q* ^2^_LOO_ = 0.79, *Q* ^2^_LMO_ = 0.78), Y-scrambling = 0.060) and external (*R* ^2^-test = 0.75) techniques, with statistical parameters confirming its robustness and applicability. Applicability domain analysis and Williams plots showed that only one outlier was present, supporting the model’s generalizability across diverse IL structures. Additionally, the model was successfully applied to predict the toxicity of previously unreported ILs, demonstrating its practical utility for guiding the design of safer, environmentally friendly ILs. Overall, this work highlights the value of combining quantum descriptors with linear modeling techniques to develop accurate, interpretable, and computationally efficient toxicity prediction models for ILs. The accuracy and generalizability of our model have shown more reliable performances in comparison to former models.

## Supplementary Material



## Data Availability

The experimental data used for training the QSAR models can be found in the Supporting Information Excel file. In this study, the COSMO-RS descriptors were taken from COSMO-RS software, and model development was performed in the QSARINS free software.

## Notes

Details of data set (Sheet 1), structural variations of cation and anion, (Sheet 2), breaking plot (Sheet 3), LMO–CV and Y-scrambling, (Sheet 4), Williams plot, (Sheet 5), descriptors values, (Sheet 6), descriptors intercorrelation. (Sheet 7), data sets matching and smiles (XLSX)
